# Epigenetic Mechanisms in Respiratory Muscle Dysfunction of Patients with Chronic Obstructive Pulmonary Disease

**DOI:** 10.1371/journal.pone.0111514

**Published:** 2014-11-04

**Authors:** Ester Puig-Vilanova, Rafael Aguiló, Alberto Rodríguez-Fuster, Juana Martínez-Llorens, Joaquim Gea, Esther Barreiro

**Affiliations:** 1 Pulmonology Department-Muscle and Respiratory System Research Unit (URMAR), IMIM-Hospital del Mar, Parc de Salut Mar, Health and Experimental Sciences Department (CEXS), Universitat Pompeu Fabra (UPF), Barcelona Biomedical Research Park (PRBB), Barcelona, Spain; 2 Centro de Investigación en Red de Enfermedades Respiratorias (CIBERES), Instituto de Salud Carlos III (ISCIII), Madrid, Spain; 3 Thoracic Surgery Department, Hospital del Mar, Parc de Salut Mar, Barcelona, Spain; University Hospital Freiburg, Germany

## Abstract

Epigenetic events are differentially expressed in the lungs and airways of patients with chronic obstructive pulmonary disease (COPD). Moreover, epigenetic mechanisms are involved in the skeletal (peripheral) muscle dysfunction of COPD patients. Whether epigenetic events may also regulate respiratory muscle dysfunction in COPD remains unknown. We hypothesized that epigenetic mechanisms would be differentially expressed in the main inspiratory muscle (diaphragm) of patients with COPD of a wide range of disease severity compared to healthy controls. In diaphragm muscle specimens (thoracotomy due to lung localized neoplasms) of sedentary patients with mild-to-moderate and severe COPD, with preserved body composition, and sedentary healthy controls, expression of muscle-enriched microRNAs, histone acetyltransferases (HATs) and deacetylases (HDACs), total DNA methylation and protein acetylation, small ubiquitin-related modifier (SUMO) ligases, muscle-specific transcription factors, and muscle structure were explored. All subjects were also clinically evaluated: lung and muscle functions and exercise capacity. Compared to healthy controls, patients exhibited moderate airflow limitation and diffusion capacity, and reduced exercise tolerance and transdiaphragmatic strength. Moreover, in the diaphragm of the COPD patients, muscle-specific microRNA expression was downregulated, while HDAC4 and myocyte enhancer factor (MEF)2C protein levels were higher, and DNA methylation levels, muscle fiber types and sizes did not differ between patients and controls. In the main respiratory muscle of COPD patients with a wide range of disease severity and normal body composition, muscle-specific microRNAs were downregulated, while HDAC4 and MEF2C levels were upregulated. It is likely that these epigenetic events act as biological adaptive mechanisms to better overcome the continuous inspiratory loads of the respiratory system in COPD. These findings may offer novel therapeutic strategies to specifically target respiratory muscle dysfunction in patients with COPD.

## Introduction

Chronic Obstructive Pulmonary Disease (COPD) is currently a major leading cause of mortality worldwide. In COPD patients, muscle dysfunction is a frequent comorbidity that imposes a substantial impact on their exercise tolerance and quality of life [1], [2]. Despite that limb muscles are usually more severely affected than the ventilatory muscles in these patients, evidence shows that respiratory muscle dysfunction also contributes to poor exercise tolerance [3]. Indeed, it has been shown that respiratory muscle dysfunction may contribute to hypercapnic respiratory failure, exercise limitation, increased risk for acute exacerbations, and eventual death in patients with COPD [4]. Several pathophysiologic factors and biological mechanisms contribute to COPD muscle dysfunction. Specifically, the increased inspiratory loads imposed by the airflow limitation and the modifications observed in thorax geometry that cause diaphragm length shortening are the main contributing factors to respiratory muscle dysfunction in COPD [3]. However, as a result of the continuous inspiratory loads overcome by the patients, the main inspiratory muscle, the diaphragm, has also been shown to experience a training-like effect, characterized by a more fatigue-resistant phenotype when compared to healthy subjects [3], [5].

Other factors such as cigarette smoke, hypoxia, hypercapnia and acidosis, exacerbations, nutritional abnormalities, and sarcopenia (aging) may also be involved in the etiology of COPD respiratory muscle dysfunction [3]. Furthermore, biological mechanisms [3] such as structural abnormalities [6], muscle remodeling [7], oxidative stress [8], and metabolic derangements [9] have also been shown to mediate respiratory muscle dysfunction in COPD. Recently, epigenetic events, defined as the heritable mechanisms that do not affect DNA sequence, have also emerged as potentially involved in the etiology of muscle dysfunction of the lower limbs in COPD patients [10]–[12], and in experimental models of disuse [13], [14] and hypertrophy [15].

Specifically, the expression of noncoding single-stranded RNA molecules, microRNAs (miRNAs) specific to skeletal muscles, was downregulated in limb muscles and blood of patients with severe COPD [10], [11]. DNA methylation is a biochemical process characterized by the addition of a methyl group to the 5 position of the cytosine that stands before a guanine molecule in the same chain. DNA methylation is the most stable modification of chromatin that may vary during development and aging in cells. Moreover, histone acetylation, defined as the balance between histone acetyltransferases (HATs) and histone deacetylases (HDACs) also seemed to regulate muscle plasticity in response to environmental factors in several models [16], [17]. Moreover, plasma levels of muscle-specific miRNAs were also shown to be increased in patients with severe COPD and peripheral muscle dysfunction [10]. Nonetheless, it remains to be identified whether differences in the activity of the muscles may influence the expression of epigenetic events. In this regard, elucidation of the epigenetic profile of the diaphragm muscle in patients with COPD has not been so far documented.

Interestingly, accumulation of small ubiquitin-related modifier (SUMO) ligases has been shown to underlie muscular dystrophies and to favor sarcopenia and muscle wasting through premature senescence of satellite cells in experimental models [18]. It remains unknown to identify whether this mechanism could also be involved in the etiology of respiratory muscle dysfunction in COPD.

On this basis, we hypothesized that epigenetic mechanisms would be differentially expressed in the main inspiratory muscle of patients with COPD of a wide range of disease severity compared to healthy controls. Accordingly, our primary objectives were to assess in the diaphragms of patients with COPD, the expression of a wide number of epigenetic mechanisms including muscle-enriched miRNAs, as well as the expression of SUMO-2/3. The existence of potential correlations between the different study variables was also analyzed in the patients. Diaphragm specimens were also obtained in healthy controls for the purpose of the investigation, and both patients and control individuals were clinically and functionally evaluated.

## Methods

(See the File S1 for detailed information on all the study methodologies).

### Study subjects

Eighteen patients with stable COPD [19]–[21] [mild (n = 9), moderate (n = 6) and severe (n = 3)] with normal body composition and 10 age-matched sedentary controls were recruited on an out-patient basis. Diaphragm muscle specimens were obtained from all subjects who underwent thoracotomy for a localized lung neoplasm.

The current investigation was designed in accordance with both the ethical standards on human experimentation in our institutions and the World Medical Association guidelines (Helsinki Declaration of 2008) for research on human beings. Approval was obtained from the institutional Ethics Committees on Human Investigation (*Hospital del Mar*, Barcelona). Informed written consent was obtained from all individuals.

### Anthropometrical and Functional Assessment

Anthropometrical, lung and muscle function evaluations were conducted as previously described [22].

### Muscle biopsies and blood samples

#### Diaphragm biopsies

During thoracotomy for localized lung lesions, diaphragm biopsy specimens were obtained from the anterior costal diaphragm lateral to the insertion of the phrenic nerve [23].

Blood samples were drawn at 8:00h am after an overnight fasting period in both patients and healthy controls.

### Molecular biology analyses

#### DNA isolation

Total DNA was isolated from vastus lateralis muscle of all study subjects using QIAmp DNA Mini Kit (QiAgen, Redwood City, CA, USA) [12], [24], following the manufacturer's protocol of DNA purification from tissues, and without the use of RNase A. Total DNA obtained from muscles was quantified using a spectrophotometer (NanoDrop, Thermo Scientific, Wilmington, DE, USA).

#### Quantification of Methylated DNA using enzyme-linked immunosorbent assay (ELISA)-based immunoassay

Global 5-methylcytosine (5-mC) in DNA was quantified in the vastus lateralis of both patients and healthy controls using MethylFlash Methylated DNA Quantification Colorimetric Kit (Epigentek, Farmingdale, NY, USA) following the precise manufacturer's instructions and previous studies [12], [25]. The minimum detectable concentration of methylated DNA in the samples was set to be 0.2 ng of methylated DNA. Data are expressed as the percentage of total methylated DNA to total DNA in the muscle samples.

#### RNA isolation

Total RNA was first isolated from snap-frozen skeletal muscles using Trizol reagent following the manufacturer's protocol (Life technologies, Carlsbad, CA, USA).

#### MicroRNA and mRNA reverse transcription (RT)

MicroRNA RT was performed using TaqMan microRNA assays (Life Technologies) following the manufacturer's instructions. 

#### Real time-PCR amplification (qRT-PCR)

TaqMan based qPCR reactions were performed using the ABI PRISM 7900HT Sequence Detector System (Applied BioSystems, Foster City, CA, USA) together with a commercially available predesigned microRNA assay, primers, and probes as shown in Tables 1 and 2 [26]. Results in the figures are expressed as the expression of fold change relative to mean value of the control group, which was equal to 1.

**Table 1:** MicroRNA assays used for the quantitative analyses of the target genes using real-time PCR.

Assay Name	Assay ID	miRBase accession number
**Muscle-specific, myomiRs**		
hsa-miR-1	002222	MIMAT0000416
hsa-miR-133a	002246	MIMAT0000427
hsa-miR-206	000510	MIMAT0000462
**Other miRNAs (highly expressed in muscles)**		
hsa-miR-486	001278	MIMAT0002177
hsa-miR-27a	000408	MIMAT0000084
hsa-miR-29b	000413	MIMAT0000100
hsa-miR-181a	000480	MIMAT0000256
		**NCBI Accession number**
U6 snRNA, housekeeping gene	001973	NR_004394

*Abbreviations*: ID, identification; hsa, homo sapiens; miR, microRNA; MIMAT, mature microRNA; snRNA, small nuclear RNA; and NR, non-coding RNA RefSeq database category.

**Table 2:** Probes used for the quantitative analyses of the target genes using real-time PCR.

Gene Symbol	Assay ID	Taqman probe context sequence (5′-3′)	Genbank accession number
EP300	Hs00914223_m1	CACCATGGAGAAGCATAAAGAGGTC	NM_001429.3
SUMO2	Hs02743873_g1	CATTGTAAAACCAAGGACAATTTTA	NM_006937.3
SUMO3	Hs00739248_m1	GCGAGAGGCAGGGCTTGTCAATGAG	NM_006936.2
GAPDH	Hs99999905_m1	GGGCGCCTGGTCACCAGGGCTGCTT	NM_002046.4

*Abbreviations*: ID, identification; EP300, E1A binding protein p300; Hs, homo sapiens; m1, multi-exonic gene assay does not detect genomic DNA; NM, mRNA RefSeq database category; SUMO, small ubiquitin-like modifier; g1, multi-exonic gene assay may detect genomic DNA if present in the sample; and GAPDH, glyceraldehyde-3-phosphate dehydrogenase.

#### Immunoblotting of 1D electrophoresis

Protein levels of the different molecular markers analyzed in the study were explored by means of immunoblotting procedures as previously described [12], [22], [23] (Figure S1 in file S1).

#### Muscle fiber counts and morphometry

On 3-micrometer muscle paraffin-embedded sections from diaphragms of the study groups, MyHC-I and –II isoforms were identified using specific antibodies as published elsewhere [22], [23].

### Statistical Analysis

Data are expressed as mean (standard deviation). Comparisons of physiological, clinical, molecular, and structural variables between the two study groups (COPD patients and control subjects) were analyzed using the Student's *t-test*. Among the patients, potential correlations between clinical, physiological and biological variables were explored using the Pearson's correlation coefficient.

## Results

### Clinical characteristics

Table 3 illustrates all clinical and functional variables of controls and COPD patients recruited in the investigation. Age, BMI, and FFMI did not significantly differ among the study subjects. COPD patients exhibited a wide range of airflow limitation from mild-to-moderate to severe disease and functional signs of emphysema compared to control subjects. Exercise capacity as measured by six-minute walking distance and cycloergometry and muscle strength as measured by maximal transdiaphragmatic pressure (Pdi_max_) and quadriceps isometric maximum voluntary contraction (QMVC) were decreased in all groups of COPD patients compared to healthy controls. Levels of fibrinogen, C-reactive protein, and globular sedimentation velocity were higher in the blood of the patients compared to control subjects.

**Table 3:** Anthropometric characteristics and functional status of all the subjects undergoing thoracotomy.

	Controls	Mild, moderate, and severe COPD
	N = 10	N = 18 (9, 6, and 3 patients)
**Anthropometry**		
Age (years)	67 (5)	69 (7)
BMI (kg/m^2^)	25 (3)	26 (3)
FFMI (kg/m^2^)	18 (1)	18 (1)
**Smoking History**		
Active, N, %	4, 40	10, 56
Ex-smoker, N, %	5, 50	8, 44
Never smoker, N, %	1, 10	0, 0
Pack/year	46 (13)	59 (30)
**Lung function**		
FEV_1_ (% pred)	81 (8)	62 (15) ***
FVC (% pred)	80 (5)	80 (14)
FEV_1_/FVC (%)	70 (5)	57 (8) ***
RV (% pred)	92 (17)	143 (40) ***
TLC (% pred)	98 (18)	101 (15)
RV/TLC	46 (6)	51 (10)
DLco (% pred)	90 (16)	69 (12) **
K_CO_ (% pred)	93 (11)	69 (17) ***
PaO_2_ (kPa)	11 (2)	10 (1)
PaCO_2_ (kPa)	5.5 (0.6)	5.3 (0.8)
**Exercise capacity**		
VO_2_ peak (% pred)	84 (10)	64 (19)***
W*R* peak (% pred)	63 (5)	46 (13) ***
Six-min walking test (m)	511 (69)	425 (101) *
**Muscle function**		
MIP (cm H_2_O)	87 (4)	82 (17)
Pdi_max_ (cm H_2_O)	134 (21)	102 (14) ***
QMVC (kg)	37 (1)	34 (3) ***
**Blood parameters**		
Albumin (g/dL)	4.1 (0.4)	4.0 (0.4)
Total proteins (g/dL)	7.1 (0.9)	6.7 (0.6)
CRP (mg/dL)	0.3 (0.2)	0.6 (0.4) *
Fibrinogen (mg/dL)	316 (23)	470 (178) **
GSV (mm/h)	6.8 (3.5)	15 (9.5) **

Values are expressed as mean (standard deviation).

*Abbreviations*: COPD, chronic obstructive pulmonary disease; N, number of patients; m, meters; BMI, body mass index; FFMI, fat-free mass index; kg, kilograms; FEV_1_, forced expiratory volume in one second; pred, predicted; FVC, forced vital capacity; RV, residual volume; TLC, total lung capacity; DLco, carbon monoxide transfer; K_CO_, *Krough* transfer factor; PaO_2_, arterial oxygen partial pressure; PaCO_2_, arterial carbon dioxide partial pressure; VO_2_ peak, peak exercise oxygen uptake; W*R* peak, peak work rate; min, minute; MIP, maximal inspiratory pressure; cm, centimeters; H_2_O, water; Pdi max, maximal transdiaphragmatic pressure; QMVC, quadriceps maximal velocity contraction; g, grams; dL, deciliter; mg, miligrams; CRP, C-reactive protein; GSV, globular sedimentation velocity; mm, millimeters; h, hour.

*Statistical significance*: *, p≤0.05, **, p≤0.01, ***, p≤0.001 between COPD patients and control subjects.

### Muscle biological markers

#### DNA methylation

**Figure 1:**
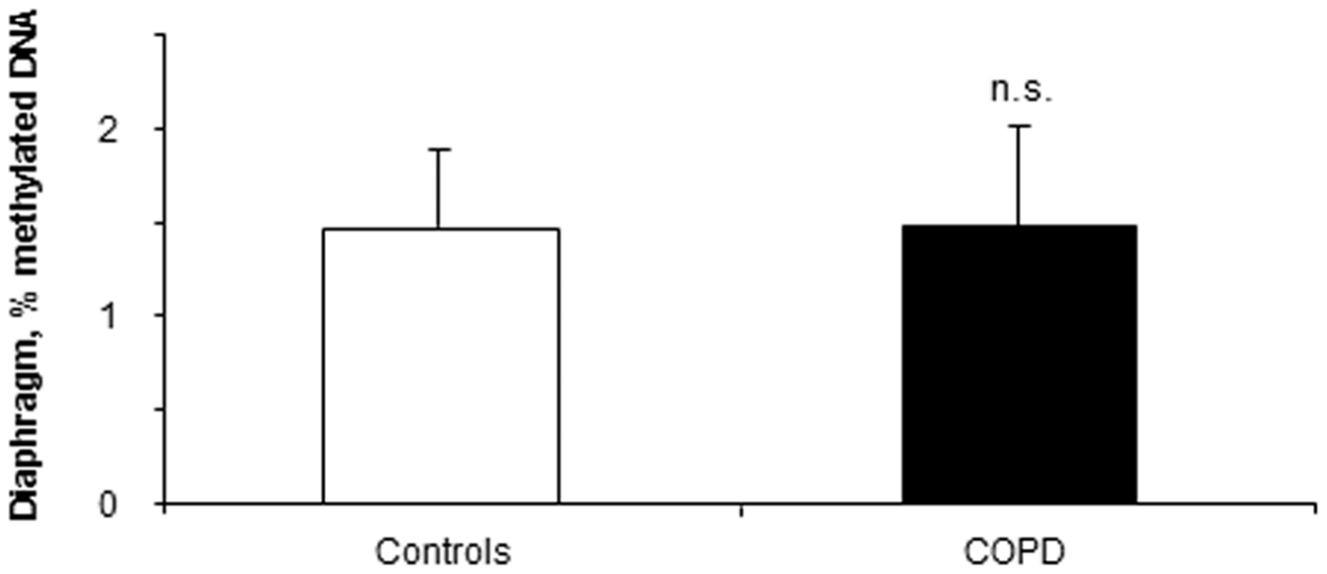
Global percentage of methylated DNA in the diaphragms of COPD patients and healthy controls. Mean values and standard deviation of global percentage of methylated DNA in the diaphragms did not differ (n.s., non-significant) between patients and controls. Samples were always run in triplicates and their corresponding expression was calculated as the mean value of the 3 measurements.

In the diaphragm, the percentage of methylated DNA to total DNA did not differ between patients and control subjects (Figure 1).

#### MicroRNAs

**Figure 2:**
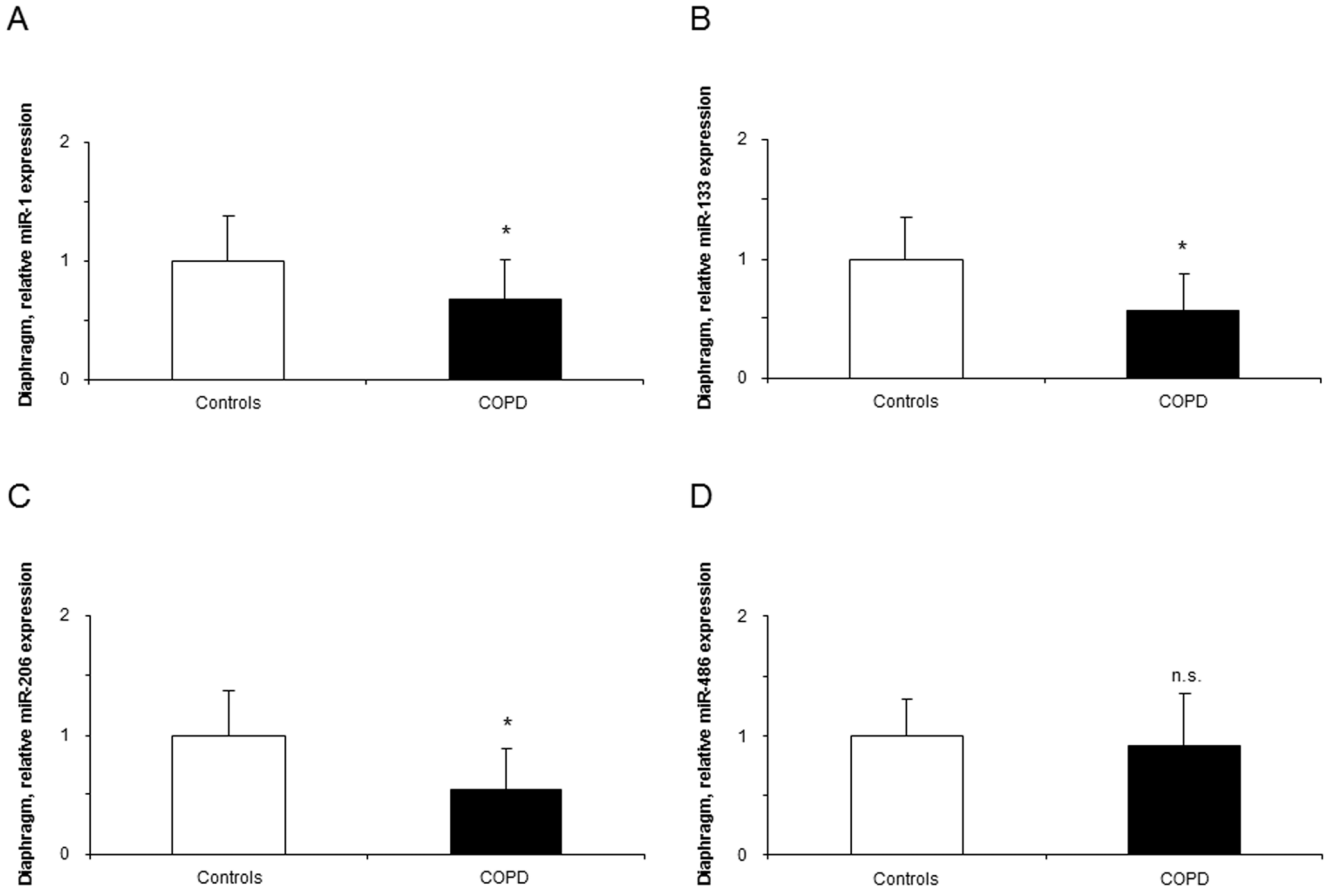
Levels of expression of muscle-enriched microRNAs in the diaphragms of COPD patients and healthy controls. Mean values and standard deviation (relative expression) of miR-1, miR-133, and miR-206 expression was downregulated (*: p<0.05) in the diaphragms of COPD patients compared to controls (panels A, B, and C), while no differences (n.s., non-significant) were detected in miR-486 expression between the study groups (panel D). Samples were always run in triplicates and their corresponding expression was calculated as the mean value of the 3 measurements.

**Figure 3:**
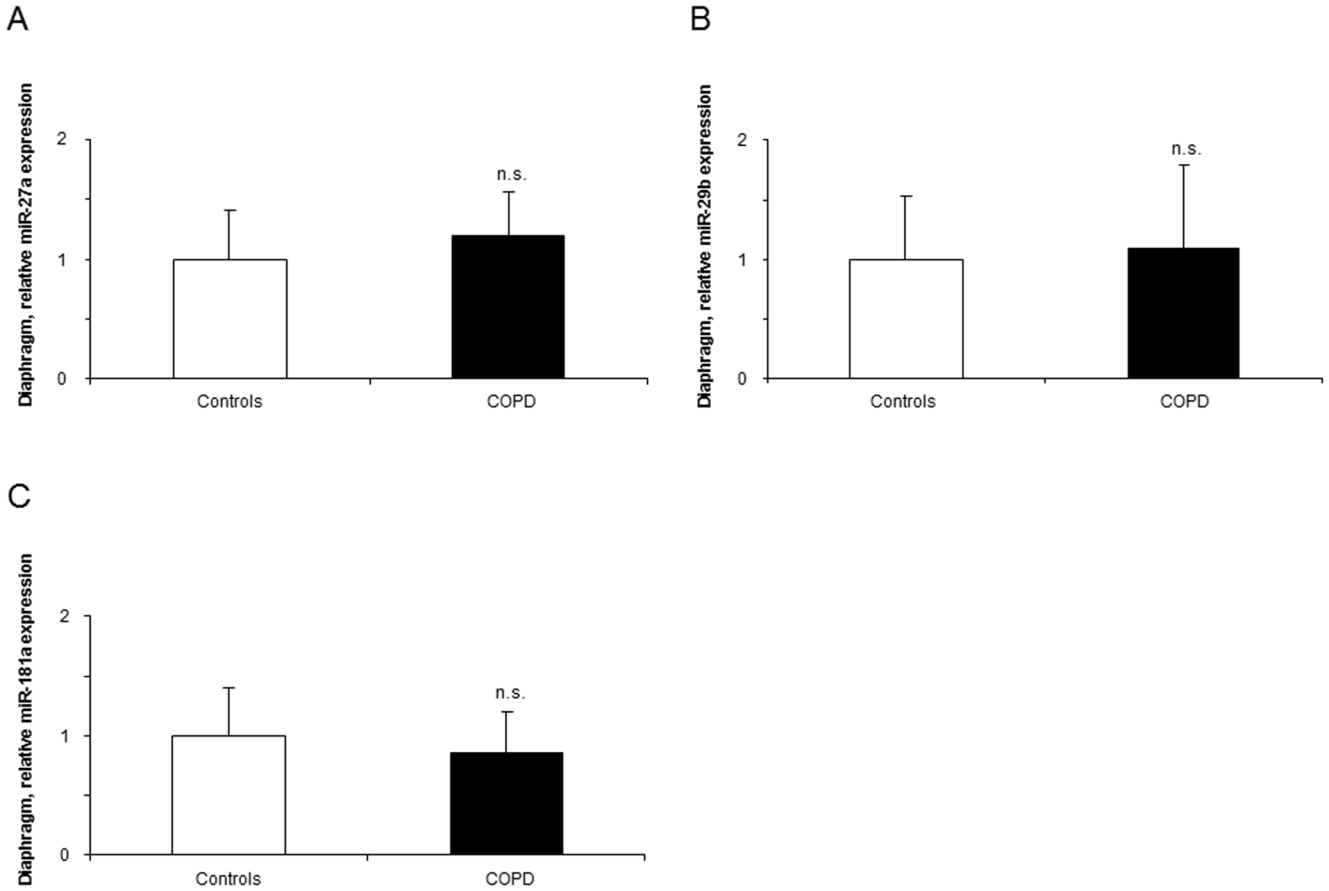
Levels of expression of muscle-enriched microRNAs in the diaphragms of COPD patients and healthy controls. Mean values and standard deviation (relative expression) of miR-27a, -29b, and -181a expression did not differ (n.s., non-significant) in the diaphragms of COPD patients compared to controls (panels A, B, and C). Samples were always run in triplicates and their corresponding expression was calculated as the mean value of the 3 measurements.

Compared to healthy controls, expression levels of miR-1, -133, and -206 were significantly downregulated in the diaphragm of the patients (Figures 2A–2C), while levels of miR-486, -27a, -29b, and -181a did not differ between the study groups (Figures 2D and 3A–3C).

#### Histone modifications

**Figure 4:**
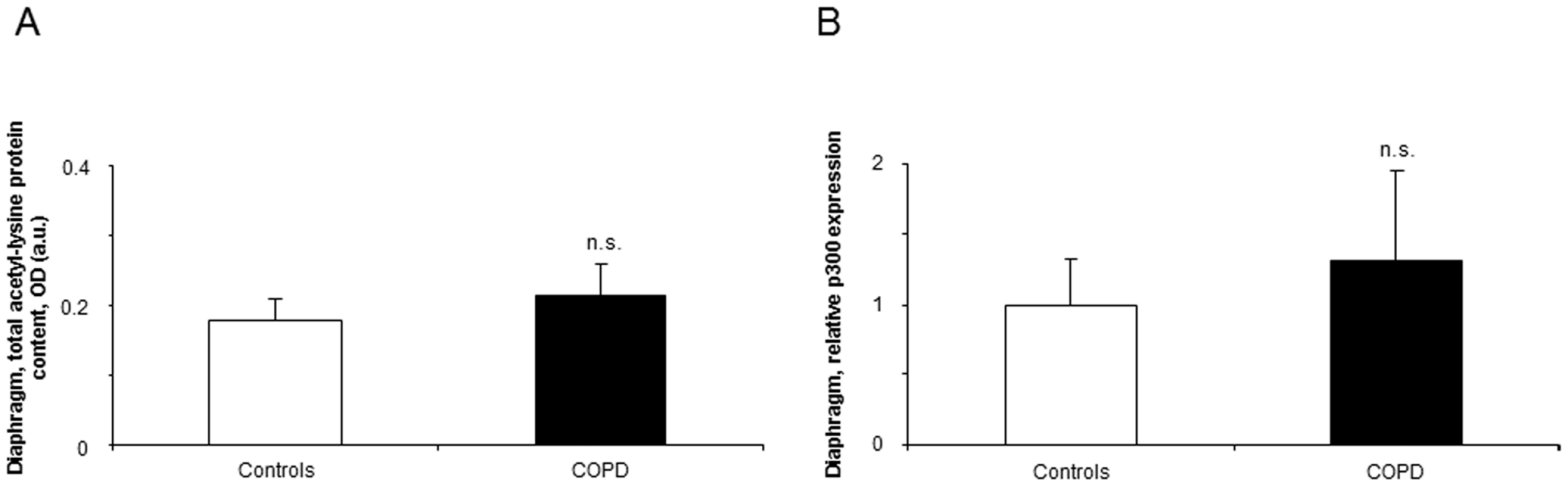
Protein levels of total acetyl-lysine protein content and mRNA levels of the HAT p300 in the diaphragms of COPD patients and controls. Mean values and standard deviation (protein levels) of total lysine-acetylated proteins and (relative expression) of nuclear cofactor p300 expression did not differ (n.s., non-significant) in the diaphragms of COPD patients compared to controls (panels A and B).

**Figure 5:**
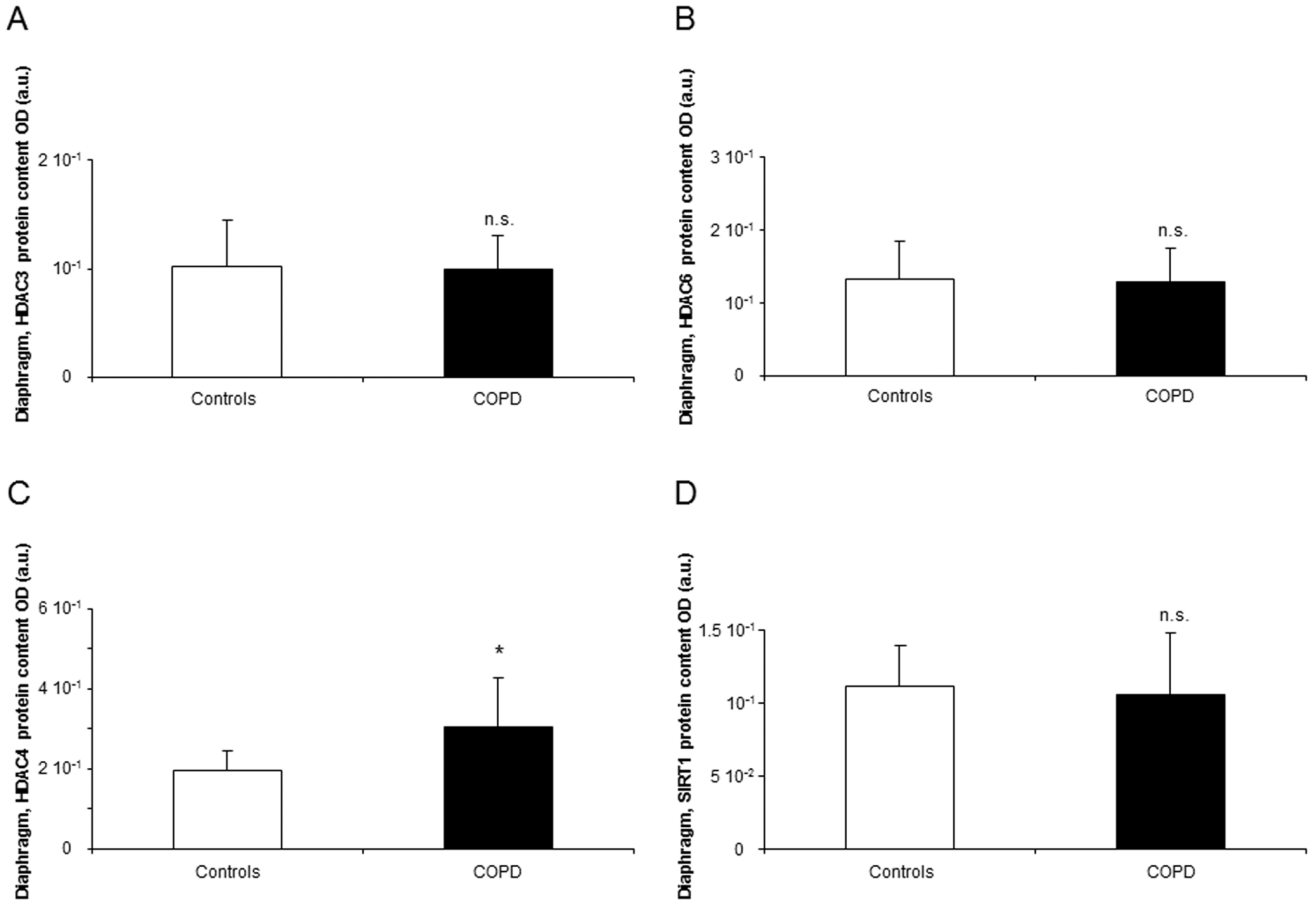
Protein levels of HDACs shown to play a role in muscle dysfunction in the diaphragms of COPD patients and controls. Mean values and standard deviation (protein levels) of HDAC3 and HDA6 did not differ (n.s., non-significant) in the diaphragms of COPD patients compared to controls (panels A and B). However, protein levels of HDAC4 were significantly greater in the diaphragms of the patients compared to the control subjects (*: p<0.05, panel C). Levels of SIRT1 did not significantly differ between patients and controls (n.s., non-significant, panel D).

In the respiratory muscle, levels of total lysine-acetylated proteins and mRNA expression levels of HAT p300 did not significantly differ between patients and controls (Figures 4A and 4B, respectively). Compared to controls, protein levels of HDAC4 were increased in the diaphragm of the patients (Figure 5C), while no differences were observed in protein levels of HDAC3, HDAC6, and SIRT1 (Figures 5A, 5B, and 5D, respectively).

#### Myogenic transcription factors

**Figure 6:**
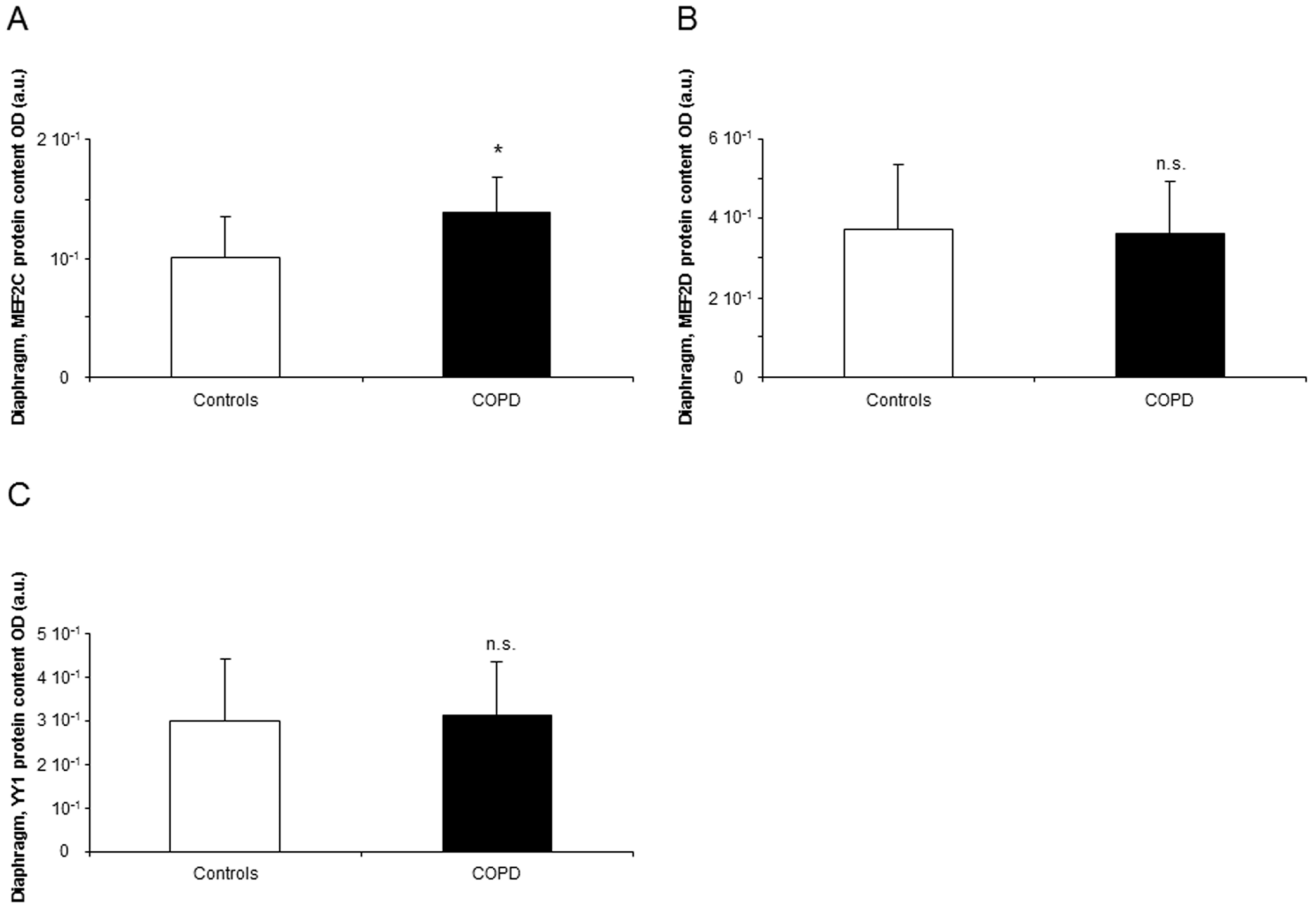
Protein levels of transcription factors shown to be involved in myogenesis and muscle repair in the diaphragms of COPD patients and controls. Mean values and standard deviation (protein levels) of MEF2C were significantly increased (*: p<0.05) in the diaphragms of COPD patients compared to controls (panel A). Nevertheless, mean values and standard deviation (protein levels) of MEF2D and YY1 expression did not differ (n.s., non-significant) between the study subjects (n.s., non-significant, panels B and C, respectively).

**Figure 7:**
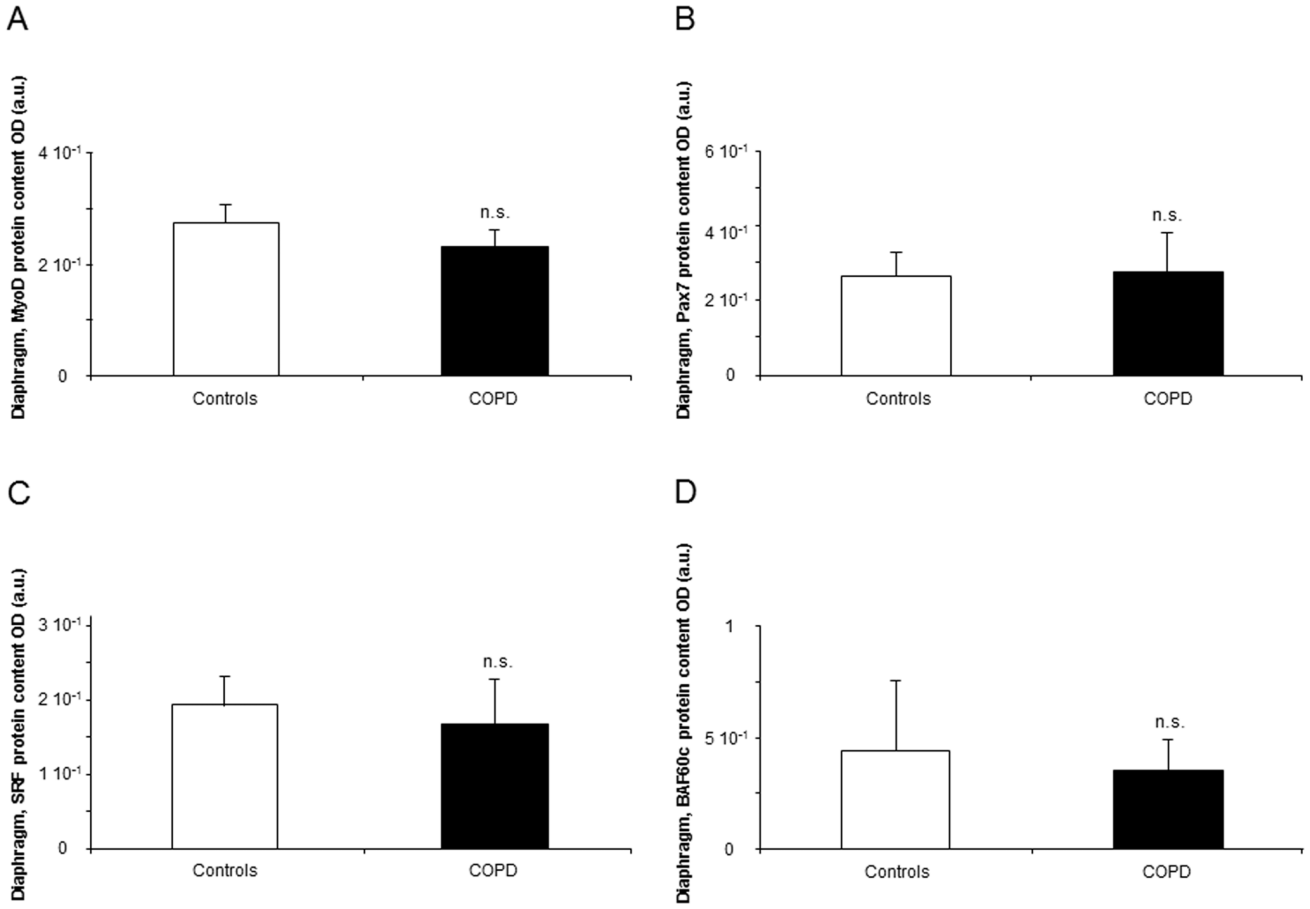
Protein levels of transcription factors shown to be involved in myogenesis and muscle repair in the diaphragms of COPD patients and controls. Mean values and standard deviation (protein levels) of MyoD, Pax7, SRF, and BAF60c expression did not differ (n.s., non-significant) between the study subjects (n.s., non-significant, panels A, B, C, and D, respectively).

Compared to controls, protein levels of MEF2C were increased in the diaphragm of the COPD patients (Figure 6A), whereas protein levels of myocyte-enhancer factor (MEF)2D, YY1, MyoD, Pax7, serum response factor (SRF) and BRG1/BRM-associated factor (BAF)60c did not differ between the study groups (Figures 6B–6C and 7A–7D).

#### Expression of SUMO

**Figure 8:**
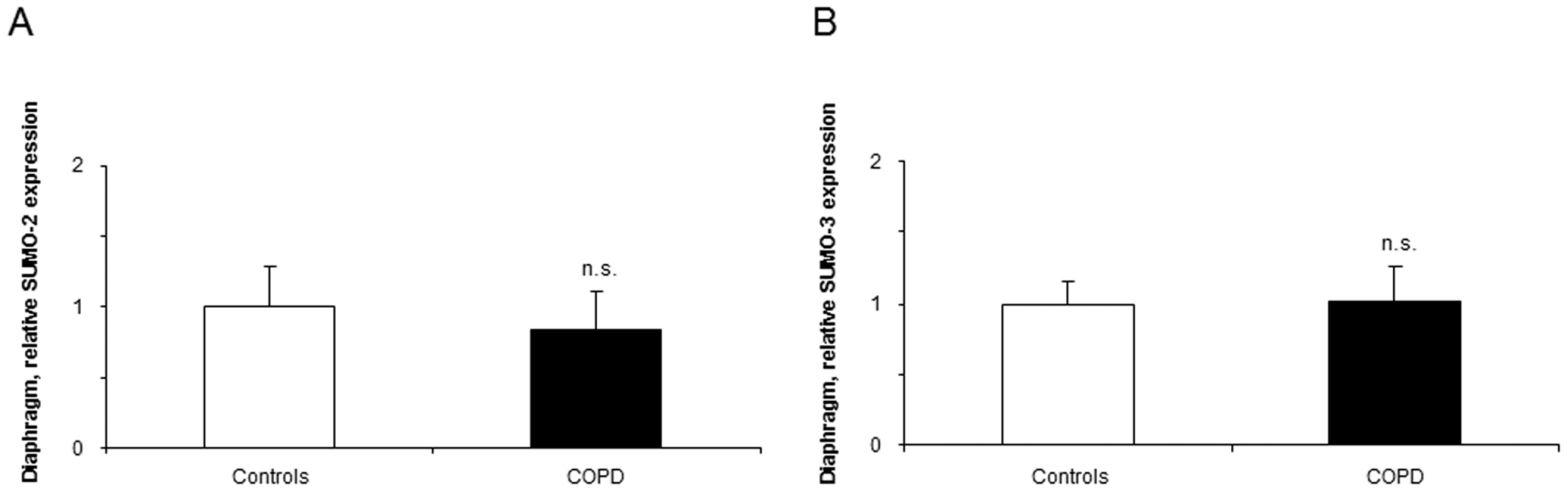
mRNA expression of SUMO-2 and SUMO-3 in the diaphragms of COPD patients and controls. Mean values and standard deviation (relative expression) of SUMO-2 and SUMO-3 expression did not differ (n.s., non-significant, panels A and B) in the diaphragms of COPD patients compared to controls. Samples were always run in triplicates and their corresponding expression was calculated as the mean value of the 3 measurements.

mRNA expression levels of SUMO2 and SUMO3 did not differ between patients and controls in the diaphragm muscle (Figures 8A–8B respectively).

### Muscle structure

#### Fiber type composition

In the respiratory muscle, no significant differences were observed in either fiber type proportions or sizes between patients and controls (Table 4 and Figure S2 in File S1). In the diaphragm of all COPD patients, an inverse correlation was found between proportions of type I fibers and FEV_1_ (r = −0.493, p = 0.038).

**Table 4:** Fiber type composition in diaphragm muscles of all the study subjects.

	Controls N = 10	Mild, moderate and severe COPD N = 18
**Muscle fiber type composition**		
**Type I fibers, percentages**	48 (5)	52 (7)
**Type II fibers, percentages**	52 (5)	48 (7)
**Type I fibers, CSA (µm^2^)**	2407 (782)	2483 (716)
**Type II fibers, CSA (µm^2^)**	2641 (713)	2410 (983)

Values are expressed as mean (standard deviation).

*Abbreviations*: CSA, cross-sectional area, µm, micrometer.

## Discussion

**Figure 9:**
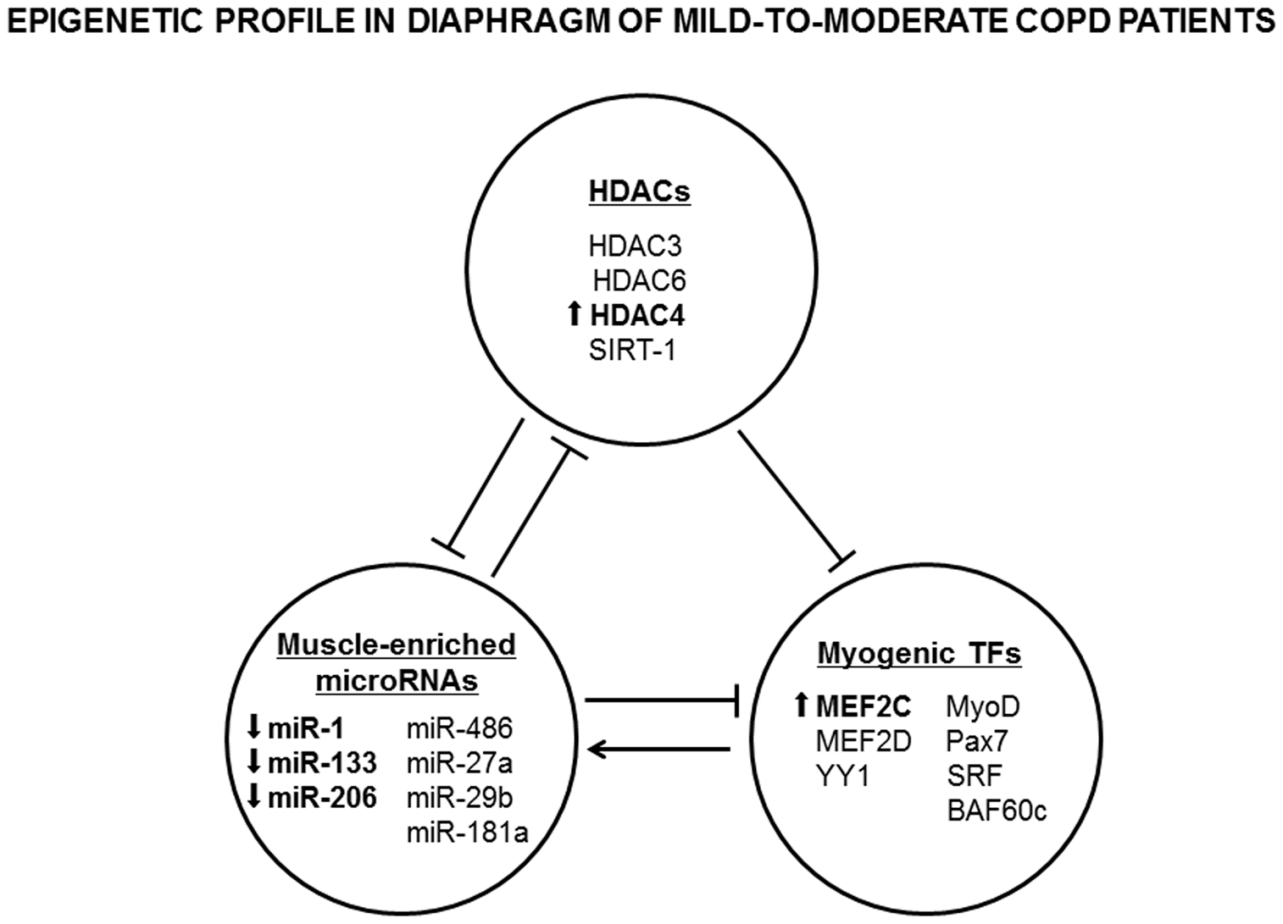
Epigenetic profile in the diaphragm of mild-to-moderate COPD patients. The main results encountered in the study are briefly depicted in the figure. Arrows express significant differences in COPD patients compared to controls. Main findings were that HDAC4 and MEF2C protein levels were increased, whereas miR-1, miR-133, and miR-206 expression levels were downregulated in diaphragms of COPD patients compared to controls. *Abbreviations:* COPD, chronic obstructive pulmonary disease; HDACs, histone deacetylases; silent mating type information regulation 2 homolog (SIRT)-1, silent information regulator 1; miR, microRNA; TFs, transcription factors; MEF, myocyte-enhancer factor; YY1, yin yang 1; SRF, serum response factor; and BAF, BRG1-associated factors.

The main findings in the current study were that in the diaphragm of patients with COPD of a wide range of airway obstruction and normal body composition compared to healthy controls, the expression of muscle-specific microRNAs such as miR-1, miR-133, and miR-206 was downregulated, while levels of miR-486, miR-27a, miR-29b, and miR-181a did not differ between the study subjects. Moreover, protein levels of HDAC4 and MEF2C were greater in the respiratory muscles of the patients than in the controls. No significant differences were observed in total protein acetylation and HAT levels, in the content of several muscle-specific transcription factors, or SUMOylation expression in the diaphragm between patients and control subjects (Figure 9). As a group, patients exhibited moderate airflow limitation, reduced diffusion and exercise capacities, as well as a moderately decrease in diaphragm force as measured by transdiaphragmatic pressure. As far as we are concerned, the present study is the first to have characterized the epigenetic profile of the main respiratory muscle of patients with COPD.

### Respiratory muscle dysfunction and structure in the COPD patients

In the present investigation, as also shown in former studies [23], [27], patients undergoing thoracotomy exhibited mild-to-moderate and severe COPD together with a moderate decrease in their exercise performance and in both respiratory and limb muscle strengths. Additionally, as also previously reported [28], [29], signs of chronic disease were evidenced by the significant rise in levels of general inflammatory parameters observed in the COPD patients. Importantly, despite that total respiratory muscle force was not reduced in the patients, the specific assessment of diaphragm strength showed a significant reduction of this parameter in that group compared to values detected in the control subjects. The degree of diaphragm muscle dysfunction seen in the patients may not be sufficiently relevant to induce a significant clinical impact at rest, but it could certainly have implications during exercise and exacerbations. In line with these findings, proportions and sizes of the diaphragm muscle fibers did not differ between patients and control subjects in the study. It is likely that changes taking place in muscle structure towards a more fatigue-resistant phenotype require a more drastic loss in muscle strength of the diaphragm, which is, in turn, directly related to the degree of the airway obstruction. In the present study, most of the patients exhibited mild and moderate airflow limitation, while only three patients had severe COPD. In previous studies from our group [23], [27] and others [7], [30], [31], respiratory muscle strength and diaphragm force parameters were more significantly reduced than in the current investigation as COPD patients were more severe. Moreover, in those investigations [7], [23], [27], [30], [31] a fiber-type switch towards a more resistant phenotype was also observed in the diaphragm of the severe COPD patients. In fact, in the study, the negative correlation encountered between the degrees of the airway obstruction as measured by FEV_1_ and the proportions of the slow-twitch fibers in the diaphragm further support these conclusions.

### Epigenetic events in the diaphragm muscle of COPD patients

Importantly, expression of the muscle-specific microRNAs miR-1, -133, and -206 was downregulated in the diaphragm of patients with mild-to-moderate and severe COPD, whereas the expression of miR-486, -27a, -29b, and -181a did not differ between patients and controls. In COPD, the inspiratory loads to which the respiratory muscle is continuously exposed may be a major player accounting for this specific pattern of microRNA expression. Aside from the classic transcription factors, muscle-specific microRNAs play a relevant role in the regulation of muscle development and repair after injury by targeting different pathways [11], [32], [33]. While miR-1 and -206 promote cell differentiation and innervation [11], [32]–[34], miR-206 induces myoblast proliferation by inhibiting myotube formation [11], [32], [33]. Recently, genetic deletion of miR-206 was also shown to delay regeneration of skeletal muscles in mice exposed to cardiotoxin injury and their myofibers were of smaller size compared to the wild type controls [35]. On this basis, it is likely that the downregulation of the muscle-specific microRNAs, especially that of miR-206 may have prevented changes in the phenotype of the diaphragm fibers in the patients, since it seems to be deeply involved in this biological process [35]. Moreover, as diaphragm muscle injury was demonstrated to be directly related to the degree of the airway obstruction and pulmonary hyperinflation in COPD [36], it could also be argued that levels of injury within the respiratory muscle fibers of the patients in this study may have been rather low to trigger miR-206 expression.

In line with the findings reported in the current study, the expression of miR-1 and -133 was also downregulated in response to muscle overload in an experimental model of hypertrophy [15]. A likely explanation to account for all these results is that the decreased expression of miR-1 and -133 may promote adaptation to overload by eliminating the posttranscriptional repression of genes and pathways involved in muscle growth such as insulin-like growth factor (IGF)-1 and SRF. In fact, protein levels of the transcription factors SRF, myoD, Pax7, and BAF60 did not differ between patients and control subjects in the study. It would be possible to conclude that the downregulation of muscle-specific microRNAs, which are part of one of the most ancient microRNA clusters, may play a crucial role in adaptation to overload in the diaphragm muscle of patients with moderate COPD.

In keeping with the findings observed in the study, spaceflight and hindlimb suspension models of atrophy also resulted in a downregulation of muscle-enriched microRNAs in the limb muscles of the exposed rodents [13], [14]. Furthermore, a pioneering study conducted on limb muscles of severe COPD and relatively preserved body composition also showed a downregulation of miR-1 among the analyzed microRNAs [11]. Furthermore, miR-1 and -499 expression levels were also positively associated with FFMI and proportions of type I fibers among the severe patients in that study [11]. Taken together, all these findings may imply the existence of a rather complex epigenetic regulation of muscle function in COPD patients than a simple up or downregulation of genes and pathways involved in muscle repair and myogenesis.

Hyperacetylation of proteins may participate in the process of muscle wasting through several mechanisms that enhance protein catabolism by ubiquitin-ligase activity of several HATs, and by dissociation of proteins from cellular chaperones [37]. Among several HATs, the nuclear cofactor p300 has been shown to regulate muscle differentiation and loss in several experimental in vivo and in vitro models [16], [38]. In the current investigation, no differences were found in p300 expression levels in the diaphragm between study groups. Differences in the study models may account for such discrepancies. Protein acetylation also relies on histone deacetylase activity. For instance, in COPD patients HDAC activity was demonstrated to be reduced in the lungs and airways of COPD patients [39]. Furthermore, in an experimental model, treatment of normal rats with the HDAC inhibitor trichostatin A also caused emphysema in the lungs of the rodents through the induction of several biological mechanisms (apoptosis and suppression of vascular remodeling) that may contribute to the perpetuation of the lung destruction [40].

In the present study, protein levels of HDAC3, HDAC6, and SIRT-1 in the respiratory did not show any significant difference between patients and controls. Nonetheless, a significant increase in HDAC4 levels was detected in the diaphragm of the patients compared to control subjects. These results are in agreement with those previously reported [11] in the vastus lateralis of patients with severe COPD and preserved body composition. Indeed, the interaction between miR-1 and HDAC4 was suggested to contribute to muscle mass maintenance, probably through insulin-like growth factor (IGF)-1 signaling [11], [41]. It should be noted that while HDAC activity and expression are downregulated in the lungs of emphysema models [39], [40], HADC4 protein levels were increased in the diaphragm of the same type of patients. Clearly, the function or the organ and type of tissue seem to strongly account for differences in the levels and pattern of expression of the different HDACs. Importantly, the findings reported herein are also in accordance with those reported in a very recent investigation [42], in which upregulation of miR-1, miR-133, and miR-206 was induced by HADC inhibition in a murine model of muscle regeneration. In view of those findings [42] and results shown in the present study, it could be argued that HADC activity is required for the upregulation of muscle-specific microRNAs expression in models of muscle injury.

Although DNA methylation plays a role in in cell fate and development, no significant differences were observed in the expression of this epigenetic marker in the diaphragm of the patients compared to the healthy controls. In light of the findings seen in the COPD patients, DNA methylation does not seem to contribute to respiratory muscle dysfunction in COPD patients, despite its potential contribution to muscle development and myogenesis.

The transcription factors myocyte enhancer factor (MEF)2 and Yin Yang (YY)1 seem to play a relevant role in muscle development, metabolic adaptation, and in the determination of muscle fiber type [43]. Interestingly, in the current study, no differences in MEF2D and YY1 were observed in the diaphragms between COPD patients and control subjects, while a significant rise in MEF2C was, indeed, seen in the patients. It is likely that in COPD, the adaptive potential is still preserved in the main respiratory muscle, at least through MEF2C upregulation.

Accumulation of SUMO ligases was shown to be associated with premature senescence of primary myogenic cultured cells [44], and other cell models [45], [46]. In this regard, sarcopenia and muscle wasting could be partly the result of the premature senescence of satellite cells, which ultimately would lead to decreased growth and repair potential. As such, premature satellite cell senescence through SUMO ligases among other mechanisms underlies the pathophysiology of muscular dystrophies [18]. In the present investigation, expression levels of SUMO-2 and -3 did not differ between groups in the diaphragm muscle, thus suggesting that premature senescence may not be a relevant mechanism of respiratory muscle dysfunction in COPD, at least in patients with mild-to-moderate airflow limitation.

### Study limitations

Diaphragm muscle biopsies from the study subjects were obtained during thoracotomy because of localized lung lesions, the gold standard technique to obtain diaphragm specimens from different populations. Although lung volume reduction surgery also makes it possible to obtain diaphragm specimens, only very severe COPD patients undergo that type of surgery, thus, making the study of mild-to-moderate patients or control subjects impossible. Therefore, diagnostic-therapeutic thoracotomy is the only approach available for studying moderate and mild COPD and normal lung function subjects. Accordingly, subjects recruited for the purpose of the study shares a common morbidity: the presence of a small and localized lung neoplasm. Nevertheless, we do not believe that this condition has made any significant contribution to the results obtained in the analyses of the diaphragm muscles, since extremely restrictive criteria were employed to properly select the population, and subjects showing either nutritional abnormalities, signs of chronic inflammation, or paraneoplastic syndromes, were systematically excluded. Therefore, we consider all the findings reported in the study to be rather associated with COPD.

## Conclusions

In the main respiratory muscle of COPD patients with a wide range of disease severity and normal body composition, muscle-specific microRNAs were downregulated, while HDAC4 and MEF2C levels were upregulated. It is likely that these epigenetic events act as biological adaptive mechanisms to better overcome the continuous inspiratory loads imposed to the respiratory system in COPD. These findings may offer novel therapeutic strategies to specifically target respiratory muscle dysfunction in patients with COPD.

## Supporting Information

File S1 Detailed methodologies. **Figure S1 in file S1:** Representative immunoblots of vinculin protein content (117 kDa) as the loading control in the diaphragms. In each fresh mini-gel, first lane from left corresponds to the molecular weight marker. All gels were always run together within the same mini-cell electrophoresis box. Definition of abbreviations: KDa, kilodaltons. **Figure S2 in file S1:** Representative immunohistochemical preparations corresponding to the staining of type II fibers in the diaphragms of a healthy control subject and a patient with severe COPD.
(DOC)
